# Quantifying the Separation Between the Retinal Pigment Epithelium and Bruch's Membrane using Optical Coherence Tomography in Patients with Inherited Macular Degeneration

**DOI:** 10.1167/tvst.9.6.26

**Published:** 2020-05-23

**Authors:** Kamron N. Khan, Shyamanga Borooah, Leonardo Lando, Kunny Dans, Omar A. Mahroo, Amit Meshi, Angelos Kalitzeos, Georgios Agorogiannis, Sasan Moghimi, William R. Freeman, Andrew R. Webster, Anthony T. Moore, Martin McKibbin, Michel Michaelides

**Affiliations:** 1 Medical Retina Service, Moorfields Eye Hospital, London, UK; 2 University College London Institute of Ophthalmology, London, UK; 3 St. James's University Hospital, Leeds, UK; 4 Department of Ophthalmology, Leeds Institute of Biomedical and Clinical Sciences, University of Leeds, Leeds, UK; 5 Jacobs Retina Center, Shiley Eye Institute, University of California San Diego, La Jolla, CA, USA; 6 Shiley Eye Institute, University of California San Diego, La Jolla, CA, USA; 7 Department of Ophthalmology, University of California San Francisco, San Francisco, CA, USA

**Keywords:** macular degeneration, inherited macular degeneration, optical coherence tomography, retinal dystrophy

## Abstract

**Purpose:**

To describe and quantify Bruch's membrane (BM) and retinal pigment epithelium (RPE) separation using spectral-domain (SD) optical coherence tomography (OCT) in patients affected by inherited macular degenerations associated with BM thickening.

**Methods:**

Patients with molecularly confirmed Sorsby fundus dystrophy (SFD), dominant drusen (DD), and late-onset retinal degeneration (L-ORD) were included in this retrospective study. Each disease was classed as early stage if subjects were asymptomatic, intermediate stage if they had nyctalopia alone, and late stage if they described loss of central vision. The main outcome was measurement of BM-RPE separation on SD-OCT. The BM-RPE separation measurements were compared against those in normal age-matched controls.

**Results:**

Seventeen patients with SFD, 22 with DD, and eight with L-ORD were included. BM-RPE separation on SD-OCT demonstrated a high test-retest and interobserver reproducibility (intraclass correlation coefficients >0.9). BM-RPE separation was not identified in normal subjects. In SFD, there was greater BM-RPE separation in late-stage disease compared with intermediate-stage patients both at subfoveal (*P* < 0.05) and juxtafoveal (*P* < 0.01) locations. In DD, there was increased BM-RPE separation in late-stage disease compared with early stage at subfoveal (*P* < 0.001) and juxtafoveal (*P* < 0.05) topographies. There was no significant difference in BM-RPE separation between disease stages in L-ORD.

**Conclusions:**

BM-RPE separation is a novel, quantifiable phenotype in the three monogenic macular dystrophies studied, and may be an optical correlate of the histopathological thickening in BM that is known to occur. BM-RPE separation, as measured by OCT, varies with stage of disease in SFD and DD, but not in L-ORD.

**Translational Relevance:**

SFD, DD, and L-ORD are associated with BM thickening. In this group of patients, OCT assessment of macular structure identifies a separation of the usually single, hyperreflective line thought to represent BM and the overlying RPE. This separation is a novel and quantifiable feature of disease staging in SFD and DD.

## Introduction

Bruch's membrane (BM) is an acellular pentalaminar matrix located between the choroid and retinal pigment epithelium (RPE), extending from the edge of the optic disc to the ora serrate.^1^^,^^2^ BM thickens with age^3^ resulting in as much as a three-fold increase in thickness at the macula.^4^^–^^8^ It has been suggested that BM thickening also contributes to the development of pathology in diseases, such as age-related macular degeneration (AMD).^9^^–^^12^

Spectral-domain (SD) optical coherence tomography (OCT) enables high-resolution structural imaging of the outer retina, BM, and choroid. In healthy eyes, four hyperreflective lines are normally seen in the outer retina. These lines have now been labeled the external limiting membrane (ELM), ellipsoid zone (EZ), RPE interdigitating zone (IZ), and the RPE-BM.^13^ Usually, SD-OCT does not demonstrate resolvable BM separation from RPE except in marked pathology.^14^^,^^15^

Abnormal BM thickening is also seen in a number of inherited diseases, including pseudoxanthoma elasticum; dominant drusen (DD), also known as Malattia Leventinese or Doyne honeycomb macular dystrophy; Sorsby fundus dystrophy (SFD); and late-onset retinal degeneration (L-ORD).^16^^–^^20^ Histopathological studies have suggested BM thickness increases with time in these conditions, and that this thickening is indistinguishable from that occurring as part of normal ageing and that accompanying AMD.^21^^–^^23^

The present study aims to characterize BM-RPE separation identified by SD-OCT in inherited macular degenerations in which BM thickening is prominent. For this study we have included patients diagnosed with SFD (OMIM 188826, 136900), L-ORD (OMIM 605670, 608752), and DD (OMIM 601548, 126600), which results from pathogenic variants in the genes *TIMP3*, *C1QTNF5*, and *EFEMP1*, respectively. These diseases are all fully penetrant and have an autosomal-dominant inheritance pattern. Currently, there are no known biomarkers of disease progression for these conditions. Here we test the hypothesis that OCT-derived BM-RPE separation can be used as a surrogate marker for BM thickening in these diseases, and that this measurement increases as the disease progresses. Additionally, we investigate whether BM-RPE separation is different in the juxtafoveal (rod-rich) region compared with the subfoveal (cone-rich) region, given phenotypic differences observed in cone- and rod-rich regions of the retina in AMD.^24^^,^^25^

## Methods

This was a retrospective cross-sectional study with patients who were identified using the inherited eye disease databases at Moorfields Eye Hospital, London, and St. James’ University Hospital, Leeds. Patients had molecularly confirmed disease causing variants in *TIMP3* (SFD), *EFEMP1* (DD), and *C1QTNF5* (L-ORD). Age-matched normal controls were identified from the inhouse database at the Jacobs Retina Center, Shiley Eye Institute, University of California San Diego, La Jolla, St. James’ University Hospital, Leeds; and Moorfields Eye Hospital, London. Eyes were excluded from analyses if there had been previous retinal surgery or a history of other chorioretinal disease. Local research ethics committee approval was obtained from Moorfields Eye Hospital, Leeds University Teaching Hospital, and the University of California San Diego sites.

**Figure 1.**
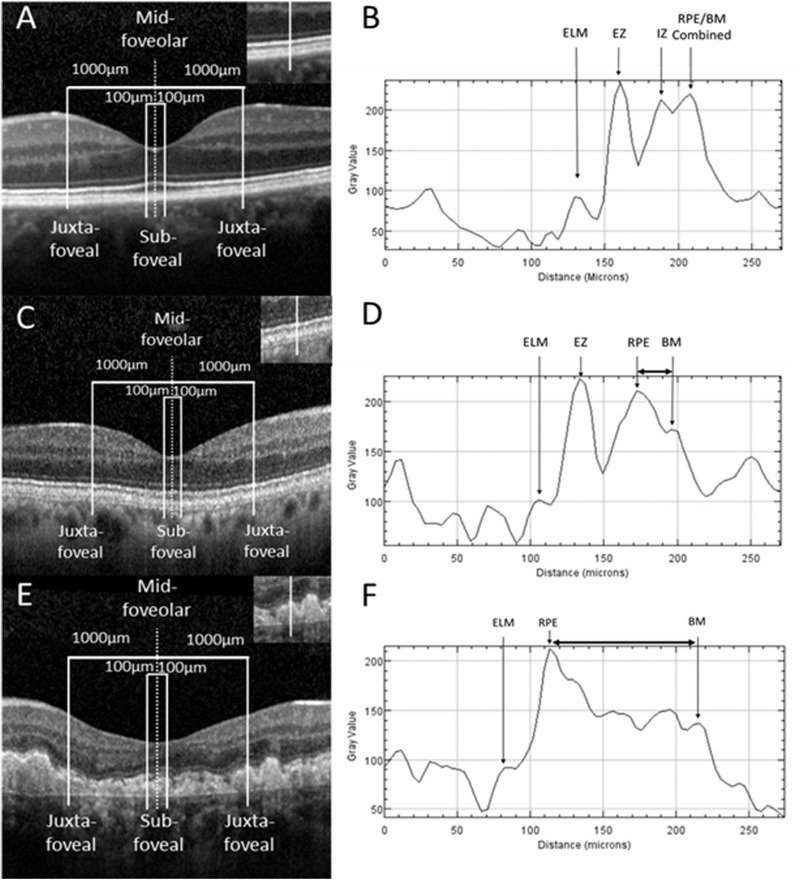
OCT scans and plot profile measurements of BM-RPE separation. Examples are shown for a normal control (A, B), an early-stage dystrophic eye with L-ORD (C, D), and a late-stage case of DD (E, F). A horizontal Spectralis SD-OCT line scan through the central fovea was analyzed for each eye of every patient and control. Readings were taken 100 µm either side of the foveola for subfoveal measurements, and 1000 µm either side of the central fovea for juxtafoveal measurements (A, C, E). A line was generated across the outer retina. The *insets* show the representative lines that were used to generate the intensity plot profiles at the farthest right juxtafoveal location (B, D, F). Intensity plot profiles across the outer retina highlight the position of the ELM, EZ, IZ, RPE, and BM. The *horizontal arrows* highlight the BM to RPE separation in the case plot profiles (D, F).

The hospital paper notes and electronic records of cases and controls were reviewed. Retinal structure was assessed using SD-OCT (Heidelberg Engineering, Heidelberg, Germany), with both line and volume scans available for interpretation. Macular OCT scans were analyzed by measuring BM-RPE separation (Fig. 1). For this, horizontal B-scans through the central fovea were selected and processed using ImageJ (National Institutes of Health, Bethesda, MD) by two members of the study team (SB and KD) using a modification of a previously published protocol.^26^

Four measurements were taken for each patient. The center of the fovea was taken as the lowest point on the B-scan judged to be closest to the fovea on the corresponding near-infrared image. Using ImageJ, the scale bar provided on the SD-OCT image was applied to scale measurements. Two subfoveal measurements in the cone-rich regions were taken (100 µm either side of the central fovea), and two juxtafoveal measurements in the rod-rich regions (1000 µm either side of the central fovea; Figs. 1A, 1C, 1E). At these four positions, an ImageJ plot profile was recorded across the outer retina (Figs. 1B, 1D, 1F). The distance between the peak hyperreflective signals (representing BM and the external border of the RPE) was noted at each site. Pixel measurements were converted to length in micrometers (µm) using the supplied scale.

**Figure 2.**
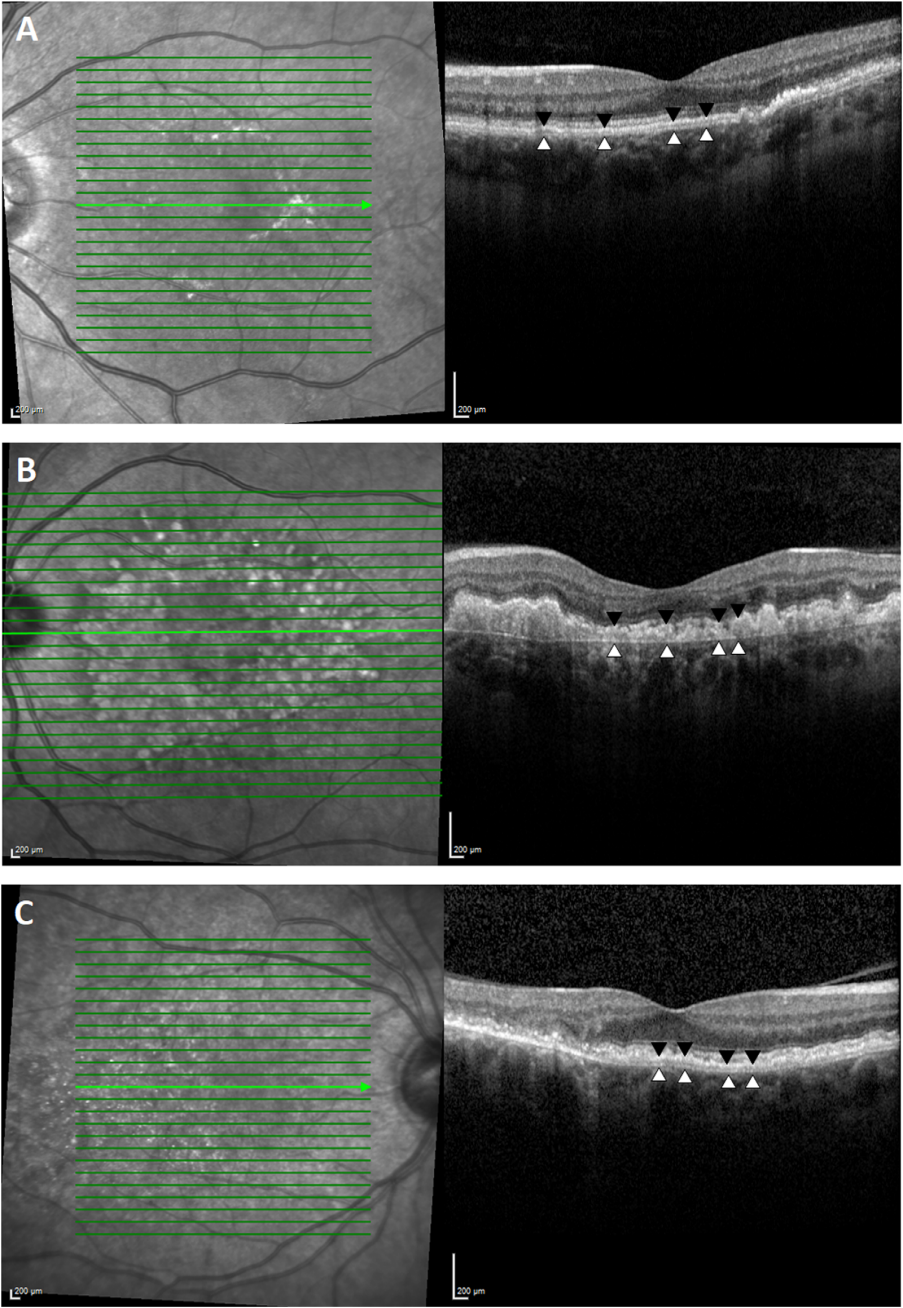
SD-OCT scans identifies distinctive separation of the RPE (*black arrowheads*) from BM (*white arrowheads*) in patients diagnosed with SFD (A), DD (B), and late-onset macular degeneration (C). The green line highlights the position of the B-scan represented in the left hand images.

Measurements of BM-RPE separation were derived from the mean distance between hyperreflective lines representing the RPE and BM, at sub- and juxtafoveal regions, measured by two independent graders, both masked to the diagnosis under analysis (Fig. 2). Case-notes were reviewed by a third investigator, and patients were grouped by disease (SFD, DD, and L-ORD). In addition, patients were classified by stage of disease: early, if they were asymptomatic; intermediate, if they reported nyctalopia alone; or late, if they described loss of central vision. For each of the inherited macular degenerations (SFD, DD, and L-ORD), BM-RPE separation measurements were compared by stage and topography (sub- and juxtafoveal regions). If the disease was asymmetric, patients were classified according to their worst eye.

Statistical analyses included generalized descriptive statistics. Measurements of BM-RPE separation were compared using a linear mixed model according to eyes and topography to enable the inclusion of data from both eyes of patients in this study. Data analyses were performed using Stata software version 15.0 (StataCorp, College Station, TX). Statistical significance for tests was set at *P* <0.05. For interrater agreement, intraclass correlation coefficients (ICCs) and their 95% confidence intervals were calculated using SPSS Statistics for Windows, Version 24.0 (IBM Corp., Armonk, NY).

## Results

A review of SD-OCT imaging of patients with inherited macular degenerations identified a separation of the usually single hyperreflective line corresponding to the BM-RPE in all three groups (SFD, DD, and L-ORD). BM-RPE separation was seen at all stages of each inherited macular degenerations, except in a single patient with asymptomatic early-stage SFD. In all the diseases, there was no significant difference in BM-RPE separation between the right and left eyes within same subject at either sub- or juxtafoveal locations.

The BM-RPE separation was measured as described earlier. Test-retest reliability of this method showed high reliability (ICC > 0.9). To see if this separation could be measured reliably using the methods described, two graders independently measured the separation at sub- and juxtafoveal locations to test interobserver reliability. ICCs showed extremely high correlation (ICC > 0.9) across all three diseases at both sub- and juxtafoveal locations. Taken together, this demonstrated that this technique could be used to reliably measure the BM-RPE separation using SD-OCT images (Supplementary Tables S1 and S2).

**Table 1.** Clinical Features of SFD

	Early Stage	Intermediate Stage	Late Stage
**Symptoms**	None	Poor vision in dark with good central vision	Poor vision in dark with reduced central vision
**Number of patients with comparative OCT**	2 eyes (1 patient)	12 eyes (6 patients)	20 eyes (10 patients)
**Mean age (SD) (years)**	39 (6.7)	45 (7.2)	47.8 (8.6)
**BM thickening**	No	Yes	Yes
**Mean subfoveal BM-RPE separation**	0 +/− 0 µm	34.4 +/− 52.4 µm	23.8 +/− 7.3 µm
**Mean juxtafoveal BM-RPE separation**	0 +/− 0 µm	69.0 +/ −47.8 µm	58.1 +/− 45.7 µm
**Stage comparison: *P*** **values**	N/A	0.042 (subfoveal) and 0.008 (juxtafoveal)	

**Table 2.** Clinical Features of DD

	Early Stage	Late Stage
**Number of individuals**	20 eyes (10 patients)	24 eyes (12 patients)
**Mean age (SD) (years)**	41 (9.7)	58 (10.7)
**BM thickening**	Yes	Yes
**Mean subfoveal BM-RPE separation**	43.0 +/− 29.1 µm	92.7 +/− 54.7 µm
**Mean juxtafoveal BM-RPE separation**	78.5 +/− 74.3 µm	108.9 +/− 57.1 µm
**Difference in BM-RPE separation (subfoveal)**	*P* < 0.001	
**Difference in BM-RPE separation (juxtafoveal)**	*P* = 0.03	

### Sorsby Fundus Dystrophy

**Figure 3.**
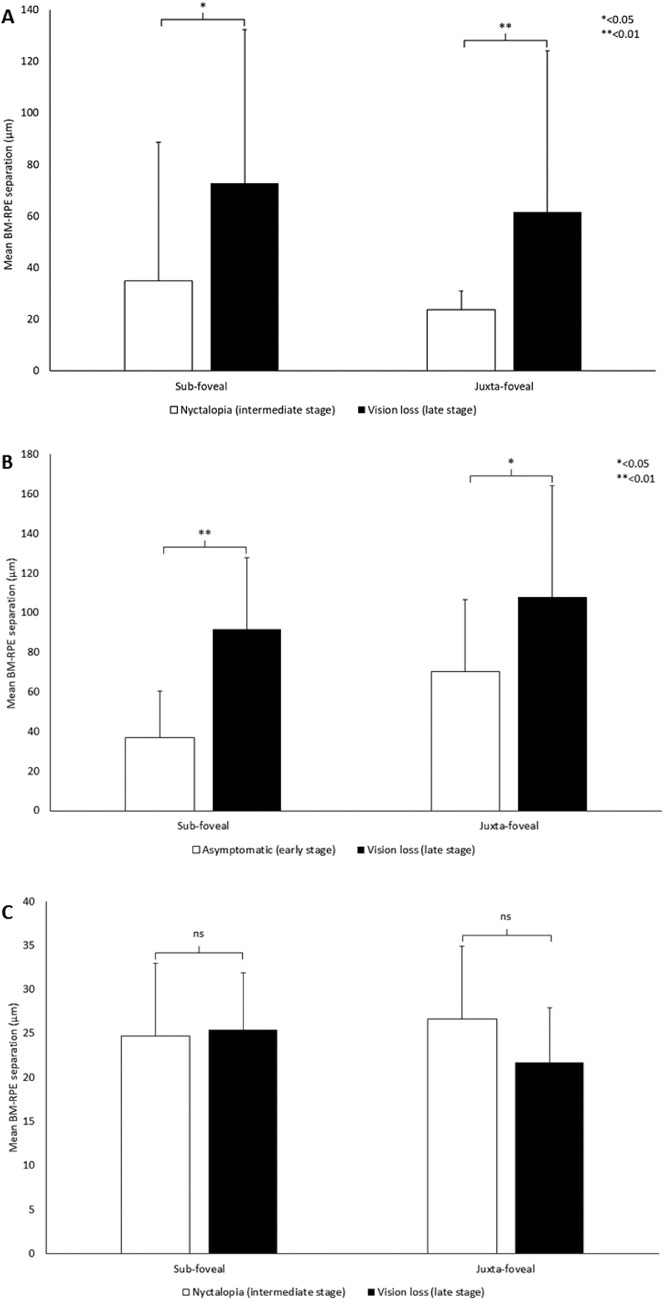
(A) Measurement of BM-RPE separation in patients with SFD. There was a significant difference in mean BM-RPE separation between patients with early-stage (n = 2 eyes) and those with intermediate-stage disease (n = 12 eyes) at both sub- and juxtafoveal loci (*P* < 0.05 and 0.05, respectively). There was also a significant increase in mean BM-RPE separation at both sub- and juxtafoveal loci as the disease progressed from intermediate- to late-stage disease (n = 20 eyes) (*P* < 0.01 and 0.01, respectively). (B) BM-RPE separation in patients with DD. The distance between BM and the RPE was significantly greater in late-stage patients (n = 24 eyes) when compared with early-stage individuals (n = 10 eyes) at sub- and juxtafoveal loci (*P* < 0.001 and 0.001, respectively). (C) Measurement of BM-RPE separation in patients with L-ORD. There appears to be no significant difference in mean BM-RPE separation between patients with intermediate-stage (n = 6 eyes) or late-stage disease (n = 10 eyes) at either sub- or juxtafoveal locations. All three graphs are shown with standard deviation bars.

Seventeen SFD patients were included in the study. These patients carried the most frequently described mutation, p.(Ser204Cys), in *TIMP3*.^27^ Only one patient (n = 2 eyes) had early-stage SFD, six (n = 12 eyes) patients had intermediate-stage disease, and 10 (n = 20 eyes) had late-stage disease by our definitions (Table 1). The early-stage case showed no discernible BM-RPE separation. Mean measurements of BM-RPE separation in intermediate SFD were 34.4 µm (52.4) at subfoveal and 69.0 µm (47.8) at juxtafoveal site. In late SFD, mean separation at respective locations was 23.8 µm (7.3) and 58.1 µm (45.7) (Fig. 3A).

BM-RPE separation was evident in all subjects with intermediate SFD and was significantly greater when compared with late stage at both sub- and juxtafoveal locations (*P* < 0.05 and < 0.01, respectively). There was no statistically significant difference in BM-RPE separation at either juxta- or subfoveal loci. No BM-RPE separation was identified in normal age-matched control individuals (n = 34 eyes), confirming a significant difference between patients with intermediate disease and age-matched controls at both sub- and juxtafoveal locations (*P* < 0.001).

### Dominant Drusen

Twenty-two patients (n = 44 eyes) with DD were identified, all resulting from the same p.(Arg345Trp) mutation in *EFEMP1*.^28^ Ten cases (n = 20 eyes) were in early-stage disease, and 12 (n = 24 eyes) in late-stage disease. No patients (n = 0) in our cohort reported isolated nyctalopia (intermediate stage) (Table 2). Mean BM-RPE separation in early DD was 43.0 µm (29.1) at subfoveal and 78.5 µm (74.3) at juxtafoveal points. At the same loci, patients with late-stage DD recorded a separation of 92.7 µm (54.7) and 108.9 µm (57.1) between OCT-derived hyperreflective lines representing BM and the RPE (Fig. 3B).

BM-RPE separation was significantly greater in patients with late-stage disease as compared with early-stage disease, at both sub- and juxtafoveal locations (*P* < 0.001 and < 0.05, respectively). In DD, separation values were significantly greater at juxtafoveal loci compared with subfoveal loci (*P* < 0.01). BM-RPE separation was significantly greater than that measured in age-matched control eyes (n = 44 eyes) at sub- and juxtafoveal locations (both *P* < 0.001).

### Late-Onset Retinal Degeneration

Eight patients (n = 16 eyes) were diagnosed with L-ORD. All cases had the most common mutation: p.(Ser163Arg) in *C1QTNF5*.^29^ No early-stage patient (n = 0) was identified for inclusion in this study. Three patients (n = 6 eyes) were identified with subtle separation of BM from the overlying RPE in the intermediate stage, and five (n = 10 eyes) in the late stage (Table 3). Mean BM-RPE separation measured in patients with intermediate-stage L-ORD was 24.1 µm (7.19) at subfoveal and 26.0 µm (9.3) at juxtafoveal locations. The same measurements in patients with late-stage disease were 25.4 µm (7.7) at subfoveal and 22.1 µm (7.1) at juxtafoveal sites (Fig. 3C).

**Table 3.** Clinical Features of L-ORD

	Intermediate Stage	Late Stage
**Number**	6 eyes (3 patients)	12 eyes (6 patients)
**Mean age (SD)**	46.8 (2.3)	67 (4.0)
**BM thickening**	Yes	No
**Mean subfoveal BM-RPE separation**	24.1 +/− 7.19 µm	25.4 +/− 7.7 µm
**Mean juxtafoveal BM-RPE separation**	26.0 +/− 9.3 µm	22.1 +/− 7.1 µm
**Stage comparison: *P*** **values**	0.761 (subfoveal) and 0.250 (juxtafoveal)	

BM-RPE separation in intermediate-stage L-ORD was significantly greater at both sub- and juxtafoveal locations when compared with normal age-matched control individuals (n = 16 eyes) (*P* < 0.001). In late-stage disease, there was marked atrophy. Using the same method to measure BM-RPE separation in patients with late disease, no significant difference was found from that recorded at the same locations in patients with intermediate-stage disease. No significant difference of BM-RPE separation for the juxta- and subfoveal sites was observed in patients with L-ORD. However, at both measured loci, BM-RPE separation was significantly greater than that observed in eyes of age-matched controls without retinal disease (*P* < 0.001).

## Discussion

BM thickening is a normal consequence of ageing, as well as being associated with several pathologic eye states.^3^^,^^5^^,^^6^ The inherited macular degenerations SFD, DD and L-ORD share histological thickening of BM as a primary pathological change, thought to result from the generation of mutant proteins, rather than haploinsufficiency.^27^^–^^29^ These proteins, all expressed by the RPE, are known to be secreted into the sub-RPE space and modulate the extracellular matrix (ECM). Although the exact disease mechanisms are not clear, studies in human postmortem samples and animal models of these inherited macular degenerations have demonstrated that either the proteins themselves, or ECM components modulated by the proteins, aggregate in the BM.^30^^–^^32^ To date, no clinical biomarkers describing the primary pathology underlying these conditions have been reported.

We initially noted an interesting phenotypic characteristic when studying SD-OCT imaging from patients with these inherited macular degenerations: a change in the hyperreflective bands in the outer retina was evident in the majority of individuals. Typically, four hyperreflective bands are visible by SD-OCT in the outer healthy retina.^15^ These bands represent the ELM, EZ, IZ, and RPE/BM complex according to an international consensus group panel.^13^ Histological correlation studies in normal retina have previously demonstrated that the outermost hyperreflective band can largely be attributed to the RPE.^15^ Unusually, here the SD-OCT scans of the patients with SFD, DD, and L-ORD demonstrated an additional separation of the fourth highly reflective outer retinal line (RPE/BM complex) into two individually resolvable reflective structures, in all but a single patient with early-stage SFD. In the majority of cases, this separation was associated with loss of a clearly identifiable IZ (Fig. 2). These features were often present at an early stage of disease, prior to visual symptoms. For patient with SFD and DD, the degree of BM-RPE separation correlated with the stage of disease: patients with the greatest ocular morbidity recorded the largest separation of BM from RPE.

BM-RPE separation, as detected by OCT, may be the optical correlate of histological thickening of BM, a feature more prominently seen in these dystrophies than is usually apparent in the aging eye. It is likely that BM/RPE complex measurements include at least some components of the RPE, as well as BM, as the highly reflective RPE melanosomes are thought to generate the innermost peak (Fig. 1).^33^ It is possible that as disease progresses, increasing RPE dysfunction is associated with progressive accumulation of basal laminar and basal linear deposit, separating the elastic lamina further from the apical RPE melanosomes and enabling the usually contiguous structures to be resolved separately. In addition, alteration in key components of BM, such as loss of heparin sulphate proteoglycans, substrates known to bind drive a parainflammatory response.^23^ Our OCT-based findings complement prior histopathology studies of postmortem donor tissue from patients with these three retinal dystrophies.^34^^–^^37^ Ex vivo microscopy studies of BM report thickening between 15 and 60 µm in samples from patients with late-stage SFD.^30^^,^^36^^,^^37^ This is similar to the mean variability and mean relative thickness recorded here using SD-OCT (Fig. 3A; Table 1).

In DD, although previous studies using SD-OCT described BM separation from RPE,^19^ precise thickness calculations have been limited by the irregular drusen.^38^ Our measurements also highlighted a wide range of values at both juxta- and subfoveal sites, which may also be related to variability in drusen accumulation (Fig. 3B; Table 2). In our patients, a significantly increased BM-RPE separation was noted at the juxtafoveal loci compared with subfoveal measurements in DD. This new finding might suggest potential similarities with AMD,^39^ a rod-driven disease that initially affects the paracentral macula.^24^^,^^25^ This difference may be influenced either by photoreceptor anatomy, or possibly regional variation in RPE structure and function, both of which change with eccentricity from the foveola.

In L-ORD, BM thickness measurements in histopathology samples from patients with advanced disease were noted to have a thickness of approximately 50 µm.^29^^,^^34^^,^^40^ Here SD-OCT measurements appear to underestimate BM relative thickness (Fig. 3C; Table 3). In patients with L-ORD, BM-RPE separation did not increase with stage of disease, possibly because one of the prominent features of L-ORD is marked outer retinal atrophy.^41^ This may highlight a limitation of using SD-OCT to estimate BM thickening in specific retinopathies associated with significant RPE atrophy, although the signal from BM can be detected, the hyperreflective band from the RPE is diminished, rendering measurements less accurate.

Other limitations of this study include the relatively small number of patients, an inherent problem when studying rare monogenic retinal dystrophies. We also recognize that measurements performed at only four sites on a single B-scan may not best represent the underlying pathogenic process, and that this could be improved by calculating the volume of the newly formed sub-RPE space bordered by the BM below. Finally, the cross-sectional design of this investigation restricts conclusions regarding progression, which needs to be addressed by long-term prospective natural history studies.

## Conclusions

BM-RPE separation is a novel, quantifiable phenotype in the three monogenic macular dystrophies studied, and may be an optical correlate of the histopathological thickening in BM that is known to occur. BM-RPE separation, as measured by OCT, varies with stage of disease in SFD and DD, but not in L-ORD.

## Supplementary Material

Supplement 1
