# Supplementary material for: Quantifying the Separation Between the Retinal Pigment Epithelium and Bruch's Membrane using Optical Coherence Tomography in Patients with Inherited Macular Degeneration
Source: Transl Vis Sci Technol. 2020 May 23;9(6):26. doi: 10.1167/tvst.9.6.26 (PMC7409156; doi:10.1167/tvst.9.6.26)
Supplement: Supplement 1 [file tvst-9-6-26_s001.docx]

Table 1. Inter-observer agreement for each disease at each location

| Disease | Topography | ICC | 95% CI | P value |
| --- | --- | --- | --- | --- |
| Sorsby fundus dystrophy | Sub-foveal | 0.936 | 0.872-0.968 | <0.001 |
|  | Juxta-foveal | 0.913 | 0.827-0.957 | <0.001 |
| Dominant drusen | Sub-foveal | 0.999 | 0.999-1.000 | <0.001 |
|  | Juxta-foveal | 0.995 | 0.992-0.998 | <0.001 |
| Late-onset retinal degeneration | Sub-foveal | 0.959 | 0.885-0.986 | <0.001 |
|  | Juxta-foveal | 0.984 | 0.955-0.995 | <0.001 |

Table 2. Test-retest agreement for each disease

| Disease | Topography | ICC | 95% CI | P value |
| --- | --- | --- | --- | --- |
| Sorsby fundus dystrophy | Sub-foveal | 0.984 | 0.963-0.995 | <0.001 |
|  | Juxta-foveal | 0.912 | 0.827-0.957 | <0.001 |
| Dominant drusen | Sub-foveal | 0.994 | 0.993-0.998 | <0.001 |
|  | Juxta-foveal | 0.999 | 0.999-1.000 | <0.001 |
| Late-onset retinal degeneration | Sub-foveal | 0.978 | 0.963-0.982 | <0.001 |
|  | Juxta-foveal | 0.903 | 0.857-0.941 | <0.001 |
